# An insight of traditional plasmid curing in *Vibrio* species

**DOI:** 10.3389/fmicb.2015.00735

**Published:** 2015-07-17

**Authors:** Vengadesh Letchumanan, Kok-Gan Chan, Learn-Han Lee

**Affiliations:** ^1^Jeffrey Cheah School of Medicine and Health Sciences, Monash University Malaysia, Bandar SunwayMalaysia; ^2^Division of Genetics and Molecular Biology, Institute of Biological Sciences, Faculty of Science, University of Malaya, Kuala LumpurMalaysia

**Keywords:** *Vibrio* species, antibiotic, resistance, plasmids, foodborne pathogens

## Abstract

As the causative agent of foodborne related illness, *Vibrio* species causes a huge impact on the public health and management. *Vibrio* species is often associated with seafood as the latter plays a role as a vehicle to transmit bacterial infections. Hence, antibiotics are used not to promote growth but rather to prevent and treat bacterial infections. The extensive use of antibiotics in the aquaculture industry and environment has led to the emerging of antibiotic resistant strains. This phenomenon has triggered an alarming public health concern due to the increase number of pathogenic *Vibrio* strains that are resistant to clinically used antibiotics and is found in the environment. Antibiotic resistance and the genes location in the strains can be detected through plasmid curing assay. The results derived from plasmid curing assay is fast, cost effective, sufficient in providing insights, and influence the antibiotic management policies in the aquaculture industry. This presentation aims in discussing and providing insights on various curing agents in *Vibrio* species. To our best of knowledge, this is a first review written discussing on plasmid curing in *Vibrio* species.

## Introduction

Antibiotic resistant infection has become more challenging to treat with existing antibiotics, leading to infections triggering higher morbidity and mortality, imposing huge costs on our society (Carlet et al., 2011; de Kraker et al., 2011; Finley et al., 2013). This increasing resistance involves many human pathogens including *Vibrio* species. *Vibrio* are Gram-negative bacteria possessing a curved rod shape that naturally inhabits the estuarine and marine environment worldwide (Hazen et al., 2010; Letchumanan et al., 2014; Raghunath, 2015). The presence of this bacterium in the marine environment raises human concern on food safety due to the latter potentially causing disease outbreaks depending on the environmental conditions (Ceccarelli et al., 2013). *Vibrio cholerae*, *V. parahaemolyticus*, *V. vulnificus*, *V. mimicus*, *V. fluvialis*, and *V. alginolyticus* are among the species commonly known to cause human illnesses (Theresa and Kumar, 2014).

The occurrence of multidrug-resistant (MDR) bacteria to clinically used antibiotics is a major health issue and a great challenge to the worldwide drug discovery programs (Alanis, 2005). It is well documented that both clinical and environmental *Vibrio* strains harbors antibiotic resistance traits (Letchumanan et al., 2015; Shrestha et al., 2015; Zavala-Norzagaray et al., 2015). A recent study in Iran has reported multidrug resistance profile towards erythromycin, sulfamethoxazole–trimethoprim and ampicillin in *V. cholerae* isolated from clinical samples (Tabatabaei and Khorashad, 2015). In India, serogroups O1 of *V. cholerae* classical biotype and sub serotype, Ogawa was identified among the *V. cholerae* isolated from clinical strains. All the isolates were reported to be resistant to ampicillin, nalidixic acid, and cotrimoxazole (Shrestha et al., 2015). Besides *V. cholerae*, *V. parahaemolyticus* have been isolated both from clinical and environmental samples study in India. A clinical study reported 178 *V. parahaemolyticus* strains were isolated from 13,607 diarrheal patients admitted in Infectious Diseases Hospital, Kolkata since 2001–2012 (Pazhani et al., 2014). Reyhanath and Kutty (2014) have reported the detection and isolation of multidrug resistant strains of *V. parahaemolyticus* isolated from a fishing land at South India. In another study, pathogenic and antibiotic resistant *V. parahaemolyticus* strains and other *Vibrio* species strains were isolated from seafood in Cochin. Majority of the strains in this study were resistant to ampicillin and multiple drug resistance was prevalent among the isolates (Sudha et al., 2014). In recent years, environmental *Vibrio* strains have been studied in detail for its potential as a reservoir for the wide spread of antibiotic resistance (Zhang et al., 2012).

Every year, more and more pathogenic *Vibrio* species have been reported to develop higher resistance towards most of the clinically used antibiotics. Drug resistance is an alarming issue worldwide and is spreading rapidly due to overuse, self-medication or the non-therapeutic use of antimicrobials (Slama et al., 2005). Antibiotics and other chemotherapeutic agents are frequently utilized in aquaculture farms as feed additives or immersion baths to achieve either prophylaxis or therapy (Devi et al., 2009; Manjusha and Sarita, 2011; Rico et al., 2012; Cabello et al., 2013). The excessive usage of antibiotics in agriculture and aquaculture environments has caused the development of multidrug resistance in seafood pathogens such as *Vibrio* species (Sudha et al., 2014). Usually the emerging of single or multiple antibiotic resistances are closely associated with various antimicrobial used (Manjusha and Sarita, 2011). Tetracycline, quinolone, oxytetracycline, enrofloxacin, sarafloxacin, and florfenicol are among the antibiotics allowed and used in the aquaculture industry to ensure continuous production of seafood (Roque et al., 2001; Yano et al., 2014).

*Vibrio* species are usually known to be highly susceptible to most clinically used antibiotics (Mala et al., 2014; Shaw et al., 2014; Letchumanan et al., 2015; Zavala-Norzagaray et al., 2015). Most of the genetic determinants that confer resistance to antibiotics are located on plasmids. Acquired antibiotic resistance in bacteria is generally mediated by extrachromosomal plasmids and transferable to other bacteria in the environment through vertical gene transfer or horizontal gene transfer (Manjusha and Sarita, 2011). Horizontal gene transfer is very important in the evolution and transmission of resistance genes between species and includes the transfer of resistance genes from fecal bacteria to environmental bacteria (Baquero et al., 2008). These extrachromosomal DNA sequences may be responsible for the emergence of resistance to multiple antibiotics (Schelz et al., 2006). In recent years, the presence of antibiotic resistance genes detected in *Vibrio* species have increased and includes β-lactam and penicillin resistance genes *penA* and *blaTEM-1* (Srinivasan et al., 2005; Zhang et al., 2009), chloramphenicol resistance genes *cat*I, *cat*II, *cat*III, *cat*IV, and *floR* (Dang et al., 2007, 2008) and tetracycline resistance genes *tet*A, *tet*B, *tet*C, *tet*D, *tet*E, *tet*G, *tet*H, *tet*J, *tet*Y, *tet*Z (Macauley et al., 2007; Zhang et al., 2009; Kim et al., 2013).

Plasmid-mediated multidrug resistance is one of the most pressing problems in the treatment of infectious diseases. The use of plasmid-curing agents may serve as a possible way to eliminate the plasmid and reduce spreading of antibiotic resistance encoded by antibiotic resistance plasmids (R-plasmids) (Molnar et al., 2003). Plasmid curing occurs naturally through cell division or by treating the cells with any chemical or physical agents (Elias et al., 2013). The inhibition of conjugational transfer of antibiotic resistance plasmid can be used to decrease the spread of antibiotic resistance plasmid in the environment. Inhibition of plasmid replication occurs in various stages and well demonstrated through the “rolling circle” model (replication, partition, and conjugal transfer). This could also be the theoretical basis for the elimination of bacterial virulence in the case of plasmid mediated pathogenicity and antibiotic resistance (Brüssow et al., 2004). The aim of this study is to provide essential insights on the traditional plasmid curing assay in *Vibrio*.

## Plasmid Curing in *Vibrio*

Bacterial plasmids are known to harbor genes for resistances to antibiotics and metals; catabolic pathways such as lactose utilization and degradation of hydrocarbons; and biosynthesis of certain antibiotics. Curing of plasmids from bacteria strains is a way to eliminate the bacteria plasmid and determine the antibiotic resistance mediation. There are several methods involving chemical and physical agents that have been developed to eliminate plasmids. Protocols for plasmid curing in *Vibrio* consist of chemical agents such as acridine orange (AO), ethidium bromide (EB), and sodium dodecyl sulphate (SDS), and physical agent (Liu et al., 2012) (**Table 1**). The mechanism of plasmid curing starts from the inhibition of plasmid replication resulted from a single nick, outside of the replication origo of the superhelical structure. The process leads to further relaxation of plasmid DNA, an increase in melting point and circular dichroism. The intercalating agents would then break the superhelical form of plasmid DNA subsequently forming an open circular or linear form plasmid DNA (Spengler et al., 2006). Resistance is usually classified as “chromosomal” when unaffected by plasmid curing and as “plasmidial” when affected.

**Table 1:** Summary of plasmid curing in Vibrio species.

No	Curing agent	[Concentration]	Media	*Vibrio* species	Results		Reference
					Before	After	
1	Sodium dodecyl sulphate	0.2–3% w/v	LBS Broth	*V. parahaemolyticus*	The isolates showed a resistance toward ampicillin, polymixin-B, streptomycin, kanamycin, neomycin, chlorotetracycline, furazolidone.	There was no apparent changes observed when susceptibility was tested against the antibiotics after plasmid curing. It indicates that the resistance to these antibiotics is found to be chromosomal.	Devi et al. (2009)
2	Acridine orange	0.100 mg/ml	Luria Bertani Broth	*V. navarrensis, V. brasiliensis, V. parahaemolyticus, V. xuii, V. coralliilyticus, V. cholerae, V. neptunius, V. alginolyticus, V. diazotrophicus, V. vulnificus B3*	Isolates were resistant to penicillin G, tetracycline, cephalothin, ampicillin, aztreonam, ceftriaxone.	Fourteen isolates with multi-resistance profile was subjected to plasmid curing. Eleven of the isolates became susceptible to the antibiotics they were resistant to after curing and three isolates were still resistant to penicillin G and aztreonam.	Costa et al. (2014a)
3	Acridine orange	0.2 mg/ml	Tryptic Soy Broth	*V. marinus, V. mimicus, V. tubiashii, V. fluvialis, V. hispanicus, V. vulnificus, V. mediterranei, V. metschnikovii, V. alginoyticus, V. harveyi*	Isolates were resistant to ampicillin, cefoxitin, oxytetracycline, nalidixic acid, tetracycline, sulfamethoxazole.	After plasmid curing, two of the isolates became susceptible to all the antibiotics they were resistant to, indicating the resistance was plasmidial. A few isolates were still resistant ampicillin after curing, suggesting it is chromosomal mediated.	Reboucas et al. (2011)
4	Acridine orange	0.05 mg/ml, 0.1 mg/ml, 0.2 mg/ml	Luria Bertani Broth	*Vibrio* species	Isolates were resistant to ampicillin, carbenicillin, cephalothin, gentamicin, netilmicin, nitrofurantoin, oxytetracycline, pefloxacin.	The strains only grew in 0.05 mg/ml AO but it was not enough to cure the strains.	Molina-Aja et al. (2002)
	Ethidium bromide	0.05 mg/ml, 0.1 mg/ml, 0.2 mg/ml	Luria Bertani Broth			All the 21 strains were cured with 0.2mg/ml EB. Six ampicillin resistant strains and one carbenicillin resistant strain became susceptible after plasmid curing	
	Sodium dodecyl sulphate	10%	Luria Bertani Broth			The treatment with 10% SDS did not cure any resistant strains.	
5	Acridine orange	0.05 mg/ml, 0.1 mg/ml, 0.2 mg/ml	Luria Bertani Broth	*V. navarrensis, V. brasiliensis, V. xuii, V. coralliilyticus*	Isolates were resistant to penicillin G, cephalothin, aztreonam, ampicillin, imipenem	All isolate resistance to be chromosomal mediated after plasmid curing.	Costa et al. (2014b)
6	Ethidium bromide	0.2 mg/ml	Tryptic Soy Broth	*V. parahaemolyticus*	Isolates were resistant to ampicillin, amikacin, kanamycin, gentamicin, cefotaxime, ceftazidime	Chloramphenicol and kanamycin resistant strains were subjected to plasmid curing. After plasmid curing, two of its chloramphenicol resistant isolate were plasmidial mediated and six were chromosomal mediated. All the kanamycin resistant strains were still resistant to the antibiotic after plasmid curing.	Letchumanan et al. (2015)
7	Ethidium bromide	0.2 mg/ml	Tryptic Soy Broth	*V. cholerae, V. parahaemolyticus, V. vulnificus*	Isolates were resistant to ampicillin, oxytetracycline, nalidixic acid.	The oxytetracycline resistance phenotype was eliminated thru plasmid curing, suggesting that the resistance to oxytetracycline was related to R-plasmids	Yano et al. (2014)
8	Ethidium bromide	0.05–0.5 mg/ml	Luria Bertani Broth	*V. mimicus, V. damsela, V. carchariae, V. metschnikovii, V. proteolyticus, V. anguillarum, V. meditteranei, V. vulnificus, V. furnissii, V. alginolyticus, V. anguillarum*	Isolates were resistant to amoxicillin, ampicillin, amikacin, co-trimoxazole, carbenicillin, cefuroxime, chloramphenicol, ciprofloxacin, chlortetracycline, doxycycline hydrochloride furazolidone, gentamycin, meropenem, nalidixic acid, netilmycin, norfloxacin, neomycin, refampicin, stretomycin, sulfafurazole, trimethoprim, tetracycline.	All of the *Vibrio* strains loss their plasmids when treated with concentration of 0.3 mg/ml EB and demonstrated a change in their resistance pattern. In their study, 79% of the *Vibrio* strains was devoid of plasmid but showed an antibiotic resistance pattern, which indicate resistance via chromosomal.	Manjusha and Sarita (2011)

### Intercalating Agents

Intercalating agents such as AO and EB have been successfully used in curing bacterial plasmids. The modes of action of intercalating agents are through preferential inhibition of plasmid replication. Basically, overnight bacteria cultures are inoculated into enrichment broths, Tryptic Soy Broth (TSB), or Luria Bertani Broth (LB). Curing agents at a concentration ranging from 0.1 to 0.5 mg/ml is added to the culture broth. The concentration depends on the organism and curing agent used. The cultures are then incubated overnight at 35 or 37°C under constant agitation. After the treatment, antibiograms assay were again performed to find antibiotic resistance phenotypes.

The effectiveness of a curing agent does vary considerably in ranging of 100- to 1000-fold. This depends on the organisms being treated, curing agent efficiency and efficacy, and the mode of action of respective the curing agent (Carlton and Brown, 1981). Due to these factors, it is essential to use a wide range of curing agent concentration especially when the bacteria are isolated from environmental sources (Trevors, 1986).

Since the 1960s, AO has been used as curing agent and normally involves loss of the whole plasmid (Salisbury et al., 1972; Costa et al., 2014a). In 1970s, Dastidar et al. (1977) reported on the efficiency of AO in eliminating R-plasmids in *V. cholerae* multidrug-resistant strains. Many other studies have demonstrated the usefulness of AO in clinical strains, animal, or environmental isolates (Kamat and Nair, 1992; Barman et al., 2010; Reboucas et al., 2011; Carvalho et al., 2013; Costa et al., 2014a) although the conventional methods for curing plasmids by curing agents may induce mutations in the host chromosomal DNA (Liu et al., 2012).

Reboucas et al. (2011) have used 0.2 mg/ml AO to cure plasmid of *Vibrio* species isolated from marine shrimp. The occurrence of multiple antibiotic resistances was observed in 29% (9/31) of *Vibrio* isolates. Of the total multi-resistant *Vibrio* isolates submitted to plasmid curing, five (55.5%) lost one or more resistance profile while four isolates (44.5%) did not lose their resistance. Out of the five isolates, two isolates became totally susceptible to all the antibiotics they were resistant to, indicating the resistance was plasmidial. Resistance to oxytetracycline was the most frequently lost phenotype after plasmid curing. The results also demonstrated that isolates were still resistant to ampicillin after plasmid curing, suggesting it could be chromosomal mediated (Reboucas et al., 2011).

Similar results were also demonstrated in another study by Costa et al. (2014a). This study subjected fourteen penicillin G, ampicillin and aztreonam resistant isolates to plasmid curing with 0.1 mg/ml of AO. After the plasmid curing assay, 11 of the isolates resistance changed from resistant to susceptible whereas, three other isolates were still resistant to penicillin G and aztreonam. Teo et al. (2000) noted the ampicillin resistant in *Vibrio* with a possible via of mediation by β-lactamase *blaVHW-1* and *blaVHH-1* genes in plasmids. AO has been used in another study by Costa et al. (2014b) and revealed the isolate resistance to be chromosomal mediated after plasmid curing. The loss of phenotype in these studies suggests that AO produce an immediate and complete inhibition of plasmid replication. However, the results may be species dependent and could not be expected with other organisms.

A study was conducted to compare the effectiveness of different plasmid curing agents. Molina-Aja et al. (2002) tested three curing treatments using AO, EB, and SDS. The strains grown in LB was tested with a set of 10% SDS; 0.05, 0.1, and 0.2 mg/ml of AO; 0.05, 0.1, and 0.2 mg/ml of EB. The results stated that the treatment with 10% SDS did not cure any of the study strains. The strains only grew in the lowest concentration of AO (0.05 mg/ml), but it was not enough to cure the stains. All the twenty-one strains were successfully treated with 0.2 mg/ml EB and seven isolates lost their plasmid. All the treated isolates presented changes in their sensitivity toward the antibiotics (Molina-Aja et al., 2002). The authors suggested that EB was selected as a favorable curing agent in comparative to AO because it is difficult to disposal of AO (Molina-Aja et al., 2002).

Ethidium bromide with a formula molecule C_21_H_20_N_3_Br is an intercalating agent which resembles a DNA base pair. Due to its unique structure, EB can easily intercalate into DNA strand. Yano et al. (2014) demonstrated the use of EB to eliminate plasmids in antibiotic resistant *Vibrio* species isolated from shrimp cultured in inland ponds in Thailand. Typically the resistant isolates were grown in TSB supplemented with 0.2 mg/ml EB. The oxytetracycline resistance phenotype was eliminated through plasmid curing. The authors suggested that the resistance to oxytetracycline was related to R-plasmids (Yano et al., 2014). Our study also utilized 0.2 mg/ml of EB in curing plasmid of *V. parahaemolyticus* isolates. The study demonstrated chloramphenicol (*catA2*) gene was detected in eight chloramphenicol resistant *V. parahaemolyticus* isolates. Two of the isolates had the gene present in their plasmid whereas another six isolates showed possibility of chromosomal-mediated since the isolates exhibit positive amplification with *catA2* gene and demonstrated phenotypic resistance to chloramphenicol on the disk diffusion test after plasmid curing. In addition, the result of plasmid curing revealed that kanamycin-resistant *V. parahemolyticus* were potentially chromosomal mediated since the isolated exhibit positive amplification with *aphA-3* gene and demonstrated phenotypic resistance after plasmid curing (Letchumanan et al., 2015).

Manjusha and Sarita (2011) performed plasmid curing using 0.05 to 0.5 mg/ml of EB. It was evident from the curing experiment that all of the *Vibrio* strains loss their plasmids when treated with concentration of 0.3 mg/ml EB and demonstrated a change in their resistance pattern. In their study, 79% of the *Vibrio* strains was devoid of plasmid but showed an antibiotic resistance pattern, which indicate chromosomal resistance. The isolates exhibited a chromosomal borne resistance toward amoxicillin, ampicillin, furazolidone, and tetracycline after curing assay. The authors concluded that some of these resistances may be encoded on plasmids in some strains, while the other isolates may be chromosomal mediated.

### Sodium Dodecyl Sulfate

Sodium Dodecyl Sulfate is an anionic detergent that is used as a chemical curing agent in *Vibrio* species. Plasmid containing cells are possibly more sensitive to SDS because of plasmid-specified pili on cell surface. The chemical acts in dislodging the indigenous plasmid from its site of attachment. Curing assay was performed using SDS as the agent in a study done in India. The *V. parahaemolyticus* isolates were inoculated into LB with different concentrations of SDS (0.2, 0.4, 0.6, 0.8, 1, 2, and 3% w/v). All the strains were still resistant to the antibiotic after curing. The study suggested that the antibiotic resistance of *V. parahaemolyticus* isolates was chromosomal borne (Devi et al., 2009).

### Physical Agents

Based on literature, physical agent such as elevated growth temperature is commonly used in *Vibrio* plasmid curing. The mode of action of elevated growth temperature is through complete or partial deletions of strain’s plasmid DNA. Elevated incubation temperature (5–7°C) above the optimal growth temperature can be used as a curing method. Study has demonstrated strains that have an optimal growth temperature of 37°C are incubated at 42°C (Carlton and Brown, 1981). The culture are incubated at the elevated temperature until it reaches late log phase, at which time it is diluted (1:20) and re-incubated at the elevated growth temperature until late log phase growth is reached again. A serial dilution is prepared and plated to obtain single colonies which are individually tested for loss of the plasmid-encoded trait and physical absence of the plasmid using agarose gel electrophoresis (Trevors, 1985).

The elevated growth temperature has been successfully used to cure tetracycline resistant, penicillinase-positive strains of *Staphylococcus aureus* (May et al., 1964). Cultures were grown at 43–44°C to obtain tetracycline-sensitive and penicillinase negative cells. However, these cells did not appear until after several cell generations at the elevated temperature. Elevated incubation temperature (up to 42°C), EB (0.5 mg/ml), 10% SDS, and AO (0.5 mg/ml) were employed to eliminate plasmids from *Vibrio* species isolated from Mai Po Nature Reserve, Hong Kong (Zhang et al., 2012). The study results stated that none of the plasmid curing agents was effective in eliminating the plasmid from host cells. All attempts to cure the plasmids from their hosts were failed, probably due to the relatively high copy number of the plasmids similar to earlier work (Zhang et al., 2007).

## Discussion

*Vibrio* species occur naturally in the aquatic environments and are normal member of the flora occurring in coastal seawater (Manjusha and Sarita, 2012). In recent years, the increasing number of emerging multi-drug resistant bacteria is distressing. The presence of antibiotic resistant genes in the bacterial plasmid have led to transmission and spreading of drug resistance among pathogenic strains. Several studies have shown conclusively that antibiotic resistance is caused by pressures of clinical antibiotics and use of antibiotics in the agricultural.

Literatures have shown bacterial resistance in *Vibrio* strains was both plasmid and chromosomal mediated. The studies have demonstrated a high incidence of antibiotic resistance against ampicillin, chloramphenicol, tetracycline, penicillin G, oxytetracycline, carbenicillin, aztreonam, cefuroxime, streptomycin, rifampicin, and amoxicillin. These are among the clinical antibiotics administrated to prevent diseases in human beings. In addition, high numbers of chromosomal mediated antibiotics resistance toward chloramphenicol and ampicillin was observed frequently in the studies. These two antibiotics along with tetracycline, chlortetracycline, nalidixic acid, gentamycin, sulfafurazole, trimethoprim are among the commonly used antibiotics in aquaculture farms through feeds during culture and hatchery production of seeds (Manjusha and Sarita, 2011). The extensive use of these antibiotics in the aquatic environments has caused the *Vibrio* species to be resistant and carry the resistant genes either in their plasmid or chromosomal. It is noted that plasmid borne integrons are the main players in being able to acquire, rearrange, and express genes conferring antibiotics resistance (Stokes and Hall, 1989; Manjusha and Sarita, 2012). These intergrons have been found in chromosomes of *Vibrio* species and many other bacteria (Heidelberg et al., 2000; Holmes et al., 2003).

The efficacy of each plasmid curing agent discussed varies depending on the concentration and the organism being cured. Based on the study’s results, it could be concluded that, EB and AO may be a better curing agent than SDS. EB was also preferred by many researchers in comparative to AO because the latter is difficult to be disposed (Molina-Aja et al., 2002). All these chemical curing agents are known to be harmful and cause health problems to human beings. Precaution steps should be followed strictly prior in handling with these curing agent during experiments. When compared with chemical curing agents, physical agent such as elevated growth temperature is least favored in *Vibrio* plasmid curing studies due to its low successful rate.

Resistance emerges either passively as an aftereffect of pre-existing innate mechanisms or actively through the acquisition of new hereditary material by mobile genetic elements for example plasmids or transposons (Summers, 2006; Wright, 2007). Traditional plasmid curing assay may be used eliminate the bacteria plasmid and detect the antibiotic resistance mediation. This vital information would be beneficial in the global surveillance management of environmental multidrug resistance. Reducing and improving the use of antibiotics in the aquatic environment can reduce resistance and allow the antibiotic to resurface eventually as an effective therapy (Barbosa and Levy, 2000). The establishment of suitable therapeutic doses of antibiotics may also help reduce potential impacts on the environment and on human health (Nogueira-Lima et al., 2006).

In summary, the paper is to provide insight to readers on the traditional plasmid curing agents its effectiveness in *Vibrio* studies. Nevertheless, a study on *Bacillus anthracis* has addressed the weakness of chemical and physical plasmid curing agents (Liu et al., 2012). The curing agents are said to cause potential mutation in the host chromosome which interferes with the functional analysis of the plasmid. For this reason, the study developed a curing method using plasmid incompatibility to study *Bacillus anthracis* plasmid (Liu et al., 2012). But this concern or approach has not been reported in *Vibrio* studies worldwide. Hence, in view of potential weakness of traditional plasmid curing agents, modern approach to plasmid curing using plasmid incompatibility or next generation sequencing (NGS) could be considered in plasmid curing of *Vibrio* studies. Another alternative approach would be using microarray technology to detect antibiotic resistant genes in bacteria (Perrenten et al., 2005; Law et al., 2015). To date, a few microarray technologies have been developed for identification of antibiotic resistance genes but are either restricted to a class of drug or limited to a certain number of genes only (Perrenten et al., 2005).

## Conclusion

To our knowledge, this is the first presentation that discuss on the traditional plasmid curing in *Vibrio* species. In the current era of science technology, traditional plasmid curing may be used to eliminate plasmids and determine antibiotic resistance mediation although there is availability of modern methods such as NGS or diagnostic displacement by specific incompatibility. Next generation sequencing has become easier to be accessed, with high throughput results and helps to locate the resistant gene in the genome. However, when compared with traditional plasmid curing, next generation sequencing involves high cost when sequencing genomes of huge samples. Usually, food safety studies involve huge number of samples thus it would be very costly to sequence all the sample isolates genome by using next generation sequencing platform. Hence, alternative approach using traditional plasmid curing is adapted by researchers. The results derived from plasmid curing assay is fast, cost effective, sufficient in providing knowledge and influence the better antibiotic management policies in the aquaculture industry. The aquaculture industry could adapt the method of switching antibiotics used in the aquatic field from time to time in order to allow withdrawal of antibiotic resistance profile in strains. As the effectiveness of clinical antibiotics has declined, the extensive use of antibiotics in the aquaculture and humans are in distress conditions due to horizontal gene transfer and spread of resistant strains. It is very crucial to deal with this threat posed by overused antibiotics in aquaculture promptly.

## Conflict of Interest Statement

The authors declare that the research was conducted in the absence of any commercial or financial relationships that could be construed as a potential conflict of interest.
